# Evaluation of cleaning and disinfection procedures on poultry farms

**DOI:** 10.1016/j.psj.2025.105453

**Published:** 2025-06-18

**Authors:** Ingrid Hansson, Tomasz Dzieciolowski, Jesper Rydén, Sofia Boqvist

**Affiliations:** a Department of Animal Biosciences, Swedish University of Agricultural Sciences, SE-750 07 Uppsala, Sweden; b Kronfågel AB, Scandistandard, SE-104 25 Stockholm, Sweden; c Department of Energy and Technology, Swedish University of Agricultural Sciences, SE-750 07 Uppsala, Sweden

**Keywords:** Broiler house, Chicken, Dip slide, Prevention, Hygiene routines

## Abstract

Proper cleaning and disinfection (C&D) of broiler houses is essential to eliminate pathogenic bacteria and minimize the risk of transmitting harmful microorganisms to subsequent broiler flocks. This study aimed to assess the effectiveness of various C&D methods, examine key factors influencing broiler house hygiene, and evaluate the impact of commonly used disinfectants.

In the first part of the study, C&D procedures were evaluated. Eighteen broiler producers collected dip slide samples after disinfection from 20 control points within a broiler house over four production rotations, resulting in a total of 1,440 samples. The second part of the study assessed the effectiveness of four commonly used disinfectants on farms with broiler houses divided into four compartments, each cleaned under the same standardized protocol. Sampling was conducted by collecting 15 dip slides from each compartment both before and after disinfection over two production rounds at each farm, resulting in a total of 720 samples.

Despite the large sample size, only a few factors significantly impacted the results. Pre-soaking surfaces with water before cleaning, combined with the use of detergents, were two improving factors. Additionally, fogging and high-pressure washing were also effective in reducing bacterial loads. To maximize bacterial reduction, disinfectants should be evenly applied across all surfaces. Neither the age nor the size of the houses showed a significant correlation with bacterial counts.

When comparing the effectiveness of different disinfectants under identical cleaning routines and broiler house conditions, no significant differences were found between the substances. However, variations were observed among the different broiler producers, with one farm showing a greater bacterial reduction, with a reduction above 400 CFU at more control points compared to the other farms suggesting that overall farm hygiene plays a crucial role.

Ultimately, effective C&D does not depend on the disinfectant but, rather, on the combined influence of all C&D variables, most importantly, on the diligence and technique of the person responsible for the process.

## Introduction

Maintaining stringent hygiene and biosecurity measures is crucial in poultry production to both prevent and control diseases. Effective cleaning and disinfection (C&D) of broiler houses is essential to eliminate pathogenic bacteria, which in turn reduces the risk of transmission of pathogenic microorganisms to subsequent flocks. Preventing disease outbreaks in poultry and transmission of zoonotic diseases through biosecurity measures, one being C&D, is considerably more effective than attempting to cure infections. One of the most prevalent diseases among poultry is colibacillosis, caused by avian pathogenic *Escherichia coli* (APEC). APEC not only compromises animal welfare but also leads to significant economic losses in the broiler industry due to decreased production, increased mortality rates, and higher condemnation rates at slaughterhouses (Nolan et al., 2013; Jonare et al., 2023). C&D should not only prevent the survival of poultry pathogens within the broiler houses; it should also prevent the survival and transmission of zoonotic bacteria, such as *Salmonella* and *Campylobacter.* A Dutch modelling study based on practical findings found that laying flocks are typically infected with *Salmonella* through the farm environment, which includes insufficiently cleaned and disinfected poultry houses (van de Giessen et al., 1994). Additionally, *Campylobacter* have been shown to survive in inadequately cleaned transport crates and be potentially transmitted to broiler flocks during thinning (Frosth et al., 2020; Hertogs et al., 2021). Similarly, *Salmonella* have demonstrated the ability to resist elimination through C&D processes with these bacteria being isolated from the slaughter line of poultry before the processing commences and contaminating the carcasses of the first slaughtered flock (Zeng et al., 2021).

Most commercial broiler producers employ an "all-in, all-out" production system, ensuring thorough C&D between flocks. There are numerous C&D protocols currently in use and the execution of them varies. However, the absence of a standardized evaluation system for these protocols necessitates the development of reliable assessment tools to verify their efficacy. The effectiveness of a disinfectant in killing microorganisms is dependent on several factors such as concentration, temperature, pH, and the residual of organic matter (Pasanen et al., 1997; Maillard, 2005). Nevertheless, no single disinfectant possesses all the desirable characteristics, such as the ability to kill pathogens, as well as being non-toxic, environmentally safe, non-corrosive, and harmless to surfaces (Maillard, 2005). A wide variety of active chemical agents are used for C&D. Although each disinfectant works through different mechanisms most involve disrupting the integrity or function of microbial cells by primarily targeting the cytoplasmic membrane and enzymes, which either prevents their reproduction or causes their death (Maillard and Pascoe, 2024). Many of these biocides can be used individually or in combination with a variety of products which vary considerably regarding their activity against microorganisms. Commercial formulations for cleaning and disinfecting broiler farms often contain highly reactive biocides with diverse chemical structures. These could include oxidizing agents that act by oxidizing proteins, lipids, and nucleotides, causing damage to the cytoplasmic membrane. Examples of these include sodium hypochlorite (NaClO), peracetic acid (CH_3_CO_3_H), and hydrogen peroxide (H_2_O_2_). An additional class of reactive biocides is alkylating agents, which react with amino acids to form crosslinks, thereby impacting enzyme function and nucleic acid structure, which results in microbiocidal effects. Examples of these include glutaraldehyde (C_5_H_8_O_2_), formaldehyde (CH_2_O), and ortho-phthalaldehyde (C_6_H_4_(CHO)_2_) (Maillard and Pascoe, 2024). There is also another class of biocides that damage cell membranes or cause a loss of membrane function. For instance, Quaternary Ammonium Compounds have surfactant properties, which disrupt cell membranes, leading to the leakage of cellular contents which impairs enzyme function and ultimately causes microbial death (Jennings et al., 2015).

A significant challenge in poultry houses is the formation of biofilms, notably within the drinking water systems. Biofilms are complex aggregations of microorganisms adhering to surfaces, encased in a protective extracellular matrix (Pascoe et al., 2015). Food-borne zoonotic bacteria, such as *Listeria monocytogenes, Salmonella* spp., and *Campylobacter* spp. have been found in biofilm on food premises (Møretrø et al., 2012). Further, *Campylobacter* has been shown to survive in biofilms in water pipes during several flock rotations due to inadequate C&D, and therefore may pose a risk of colonization for the subsequent chicken flocks. (Battersby et al., 2016; Ferrari et al., 2019; Frosth et al., 2020). Bacteria within biofilms exhibit an increased resistance to antimicrobials and disinfectants, making their eradication challenging (Shree et al., 2023). Biofilms are also known to be difficult to remove with C&D. For example, Stoller et al. (2019) found that strains of *L. monocytogenes* could persist for four years within a meat processing facility and they exhibited resistance to peracetic acid, a commonly used disinfectant at farm level, slaughter houses, and food processing facilities. Therefore, implementing effective strategies to prevent and remove biofilms is vital for inhibiting the survival of pathogenic bacteria and maintaining good water quality, and selecting an optimal disinfectant is a crucial aspect to this.

The aims of the study were to: (i) evaluate the effectiveness of various C&D protocols on broiler farms, (ii) examine the influence of factors of broiler farms such as the size and age of broiler houses, and (iii) assess the impact of the most frequently used disinfecting agents in cleaning and disinfecting the broiler houses.

## Material and methods

### Cleaning and disinfection procedures in broiler houses

Throughout Sweden, around 110 million broiler chickens are produced annually, and of these around 98 % are conventionally raised by members of the Swedish Poultry Meat Association. This association is responsible for establishing the rules and guidelines for chicken production and encompasses the entire broiler production chain, including breeding companies, hatcheries, feed manufacturers, farmers, and slaughterhouses. All conventionally reared broilers in Sweden are produced through an all-in-all-out system, which includes thorough cleaning between each production round. However, the specific procedures and chemical substances used for C&D vary among different producers as there are no detailed guidelines. On certain farms, the farm’s own personnel perform the C&D, while on others, it is outsourced to contractors. The cleaning process commences immediately after all broilers in the flock have been delivered to slaughter. The first step involves the removal of all litter, feed residues, and organic material from the broiler house using a loader. Dust and dirt are then blown down from all surfaces towards the floor, after which the floor is swept, generally with a cylinder broom. In some broiler houses, surfaces are soaked with water before the main wash, which is performed with a high-pressure washer. The main wash may involve cold or warm water and, in some cases, detergent to clean all surfaces in the broiler house. Once the surfaces have dried, most producers apply a disinfectant and thereafter prepare the house for the next flock. No specific testing is required to assess the effectiveness of their C&D procedures.

### Study design

The study was conducted in two parts. The first part (hereafter referred to as ‘*Cleaning of broiler houses*’) evaluated the C&D procedures on broiler farms, and the second part (hereafter referred to as ‘*Comparison of disinfectants*’) assessed the effectiveness of some of the most used disinfections at the included farms. Initially, 25 broiler farms from a district in the south of Sweden with 40 broiler producers were selected to be included in the first part of the study. All selected farms used an all-in all-out production system with C&D between flocks. Chickens had free access to water and feed through automatic water and feed lines. The ventilation systems were either negative-pressure or neutral-pressure. All broiler houses featured polished concrete floors and an anteroom where workers changed clothes and shoes before entry. The selection criteria stated that there should be an even geographical distribution, that the farms were representative of Swedish broiler production, and that the farmer was willing to participate. At each broiler farm four production rounds were included. To ensure that the data collection covered all seasons, sampling was performed over a 14-month period from January 2020 to March 2021.

The second part of the study, *’Comparison of disinfectants’*, was conducted on three broiler farms from the first study, during two production rounds. The inclusion criteria for the selected farms for this part of the study stated that each farm contained at least one house consisting of four identical broiler compartments and that the farmer was willing to participate. All four compartments within each house were cleaned according to the same cleaning protocol. After cleaning, each compartment was disinfected using one of the three commonly used disinfectant preparations: hydrogen peroxide, peracetic acid, or glutaraldehyde combined with benzalkoniumcloride. Additionally, chlorine dioxide, a disinfectant not previously used by any of the farmers, was tested. Chlorine dioxide is considered environmentally friendly due to its easily degradable ingredients which do not accumulate in nature. All disinfectants in the study ‘*Comparison of disinfectants’* were applied using a cold fog resonator, which was selected because chlorine-based disinfection could not be heated. The cold fog resonator was powered by electricity, which prevented the emission of combustion gases and produced fine disinfectant droplets, up to 8 microns in size, enhancing the coverage on surfaces. This method ensured uniform application across all farms, thereby reducing potential variability in disinfection efficiency due to differences in application techniques. The use of small droplets also helped to increase surface contact with the disinfectant which optimized the disinfection process.

### Sampling and bacteriological analyses for the ‘Cleaning of broiler houses study’

Sampling was performed immediately before the preparation for the subsequent flock, which should be at least three days after disinfection was completed. Eighteen of the 25 polled broiler producers collected dip slide samples from 20 control points in a broiler house distributed across eight different surfaces with one to four samples from each surface at four different occasions. This resulted in a total of 1440 samples across the 18 farms (18 broiler farms x 20 control points x 4 sampling occasions = 1440 samples) (Table 1). The control points were based on the research teams’ experience of critical control points as well as a study performed on broiler farms in Belgium (Maertens et al., 2018). The sampling was performed by the farmer using double-sided dip slides (Envirocheck® Contact TVC, Merck, KGaA, Darmstadt, Germany) coated with nutrient agar with a total surface area of 19 cm². Both sides of each dip slide were pressed against the sampling point, then placed back into their plastic containers, and sent in padded envelopes together with a filled questionnaire containing data regarding cleaning routines at that production round. Once these arrived to the bacteriological laboratory at the Department of Animal Biosciences, Swedish University of Agricultural Sciences (SLU) the analyses began within 24 hours of sampling. The dip slides were incubated in an upright position at 37 ± 1 °C and examined for growth after 48 ± 4 hours. The approximate number of visible bacteria (colony-forming units, CFU) was counted on both sides of the dip slides. Colonies with different appearances (such as molds or yeasts) were excluded from the count, ensuring that only relevant bacterial colonies were considered in the analysis.

**Table 1:** Control points sampled at surfaces in broiler houses evaluating the cleaning procedures.

Surface	No. of samples	Description of control points
Floor	3	1st one meter from the entrance, 2nd in the center, 3rd at the end of the broiler area
Walls	3	30 cm above the floor at a corresponding level to the floor samples
Ceiling	2	Directly above the 1st and 3rd floor samples
Air inlets	2	Closest air inlet at corresponding level to the other samples
Feed container	1	Inside the feed container
Waterlines	4	Outer surface of waterlines and close to the floor samples
Anteroom	1	Floor of the anteroom, one meter outside the entrance of the broiler area.

### Sampling and bacteriological analyses for the “Comparison of disinfectants study”

Fifteen samples were collected from each broiler compartment by the broiler producers before disinfection and 15 after disinfection using PCA (TTC) + *N* contact slide (Liofilchem, Abruzzi, Italy). Samples were collected from three specific surfaces within each compartment: the floor (*n* = 5), walls (*n* = 5), and inner ceiling (*n* = 5). This resulted in a total of 720 samples across the three farms (3 farms × 2 sampling occasions × 2 (before and after) × 15 samples = 720 samples. The incubation of the contact slides was initiated within 24 hours at 30± 1 °C for 72 ± 4 hours, with examinations conducted every 24 hours to examine and quantify bacterial growth.

### Epidemiological data collection

A written questionnaire was sent to the broiler producers at each sampling occasion in the ‘*Cleaning of broiler house’* study. The questionnaire contained both qualitative and quantitative questions regarding the broiler houses, as well as their C&D routines (Table 2). The questionnaires were completed by the broiler producer and subsequently sent to the laboratory along with the collected samples.

**Table 2:** Information collected through a questionnaire focusing on cleaning routines in the ‘*Cleaning of broiler houses study’*.

Variable	Categories
• Age of the broiler house	Year
• Size of the broiler house	Length*Height*Width (m)
• Duration of empty period	Days
• Was the broiler house soaked with water before cleaning	Yes/No
• Was a detergent used in connection with cleaning	Yes/No
• If yes, in previous question which detergent	Open question
• Temperature of water used for cleaning	Cold/Lukewarm/Warm
• Time between cleaning and disinfection	Hours/Days
• Was disinfection used after cleaning	Yes/No
• If yes, in previous question which disinfection product	Open question
• Application method for disinfection	High pressure/Fogging/ Other

### Statistical analysis

The arithmetical mean of the obtained bacterial colony counts from both sides of the dip slides for each sampling occasion were used for statistical analyses. The data derived from the questionnaires and corresponding arithmetical means of the bacterial counts were used to generate a database (Excel, Microsoft Office 2010). To determine any possible impact of the independent variables on the dependent variable (arithmetical means of the bacterial counts) models for multiple regression were employed with farm acting as a block variable. The statistical analysis was carried out with R (R Core Team, 2025).

## Results

### Cleaning of broiler houses

**Fig. 1:**
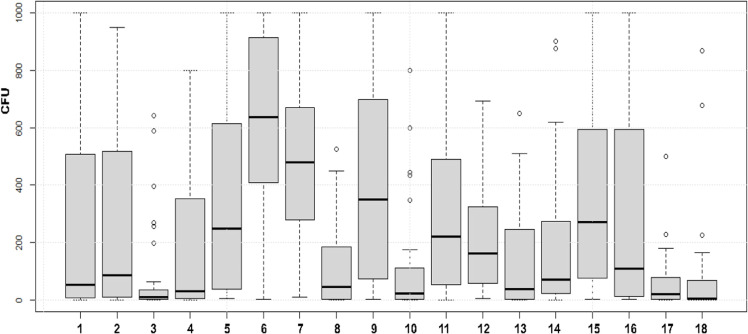
Distribution of bacterial counts, expressed as colony forming units (CFU) per dip slide sampled after cleaning and disinfection at 20 control points across four production rounds at 18 different broiler farms. Boxes show values between the 25th and 75th percentiles with the median indicated by a line. Values below the 10th and above the 90th percentile are shown as circles.

**Fig. 2:**
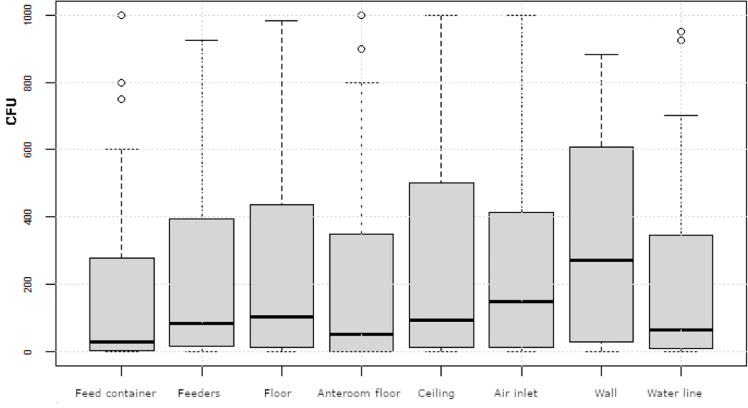
Distribution of bacterial counts, presented as colony forming units (CFU) per dip slide collected at eight different surfaces, with each surface receiving one to four control points at 18 farms after cleaning and disinfection at four production rounds. Boxes show values between the 25th and 75th percentiles with the median indicated by a line. Values below the 10th and above the 90th percentile are shown as circles.

This study involved eighteen broiler producers who collected dip slide samples at four production rounds. The age of the poultry houses ranged from 3 to 41 years, with a mean of 14 years, and the size of the houses were 1.944 m^3^ to 11.378 m^3^ with a mean of 7.526 m^3^. The flock density was 36 kg/m^2^ in all broiler houses, with the number of chickens per flock ranging from 16 000 to 72 000, and a mean of 43 000. Neither the age nor the size of the broiler house was associated with the number of bacteria remaining after C&D. However, there was a substantial variation in bacterial counts between different farms (*P* < 0.001) (Fig. 1). The floor in all broiler houses consisted of polished concrete and the walls in one of the farms, Farm 10, consisted of plywood whereas the walls in the rest of the houses were made of concrete. All participating farms except one, Farm 6, used detergent during cleaning. The farms that did not use detergent had the highest bacterial load, with a mean above 600 CFU, which could be compared to some of the other farms with a mean below 100 (Fig. 1). Nevertheless, no difference in bacterial counts between the different sampling surfaces in the broiler houses was observed (Fig. 2).

**Fig. 3:**
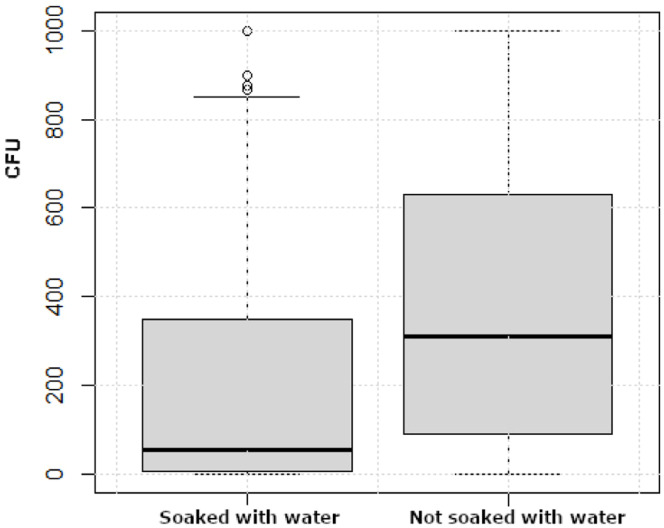
Distribution of bacterial counts presented as colony forming units (CFU) in dip slide samples from 1440 control points sampled during 72 production rounds at 18 farms categorized by broiler houses soaked with water (*n* = 57) and not soaked with water (*n* = 15) before cleaning. Boxes show values between the 25th and 75th percentiles with the median indicated by a line. Values below the 10th and above the 90th percentile are shown as circles.

The empty period between two flocks varied from 7 to 22 days (mean 12 days) and the time between C&D from 3 hours to 14 days (mean 3 days). Neither the duration of the empty period nor the time between C&D correlated with bacterial counts remaining after C&D. Majority of the farmers (80 %) soaked the surfaces in the broiler house before cleaning. A difference (*p* < 0.001) was observed regarding the bacterial count in samples from broiler houses soaked with water before cleaning compared with those that were not soaked with water before cleaning (Fig. 3).

**Fig. 4:**
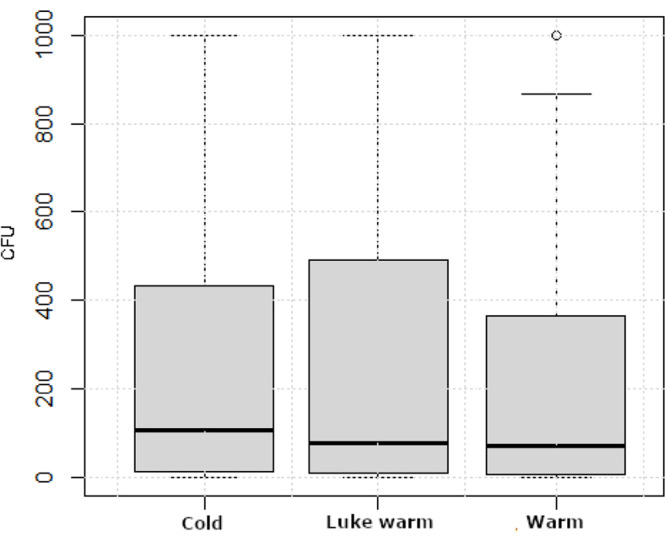
Distribution of bacterial counts presented as colony forming units (CFU) in dip slide samples from 1440 control points sampled during 72 production rounds at 18 farms categorized based on whether water used during cleaning was cold (*n* = 46), lukewarm (*n* = 10), or warm (*n* = 16). Boxes show values between the 25th and 75th percentiles with the median indicated by a line. Values below the 10th and above the 90th percentile are shown as circles.

Cold water was mostly (64 %) utilized to clean the broiler houses, likely due to both the access and cost of heating the water. However, no differences were found in bacterial load in the surfaces of the broiler houses after C&D, regardless of whether the water used during cleaning was cold, lukewarm, or warm (Fig. 4).

**Fig. 5:**
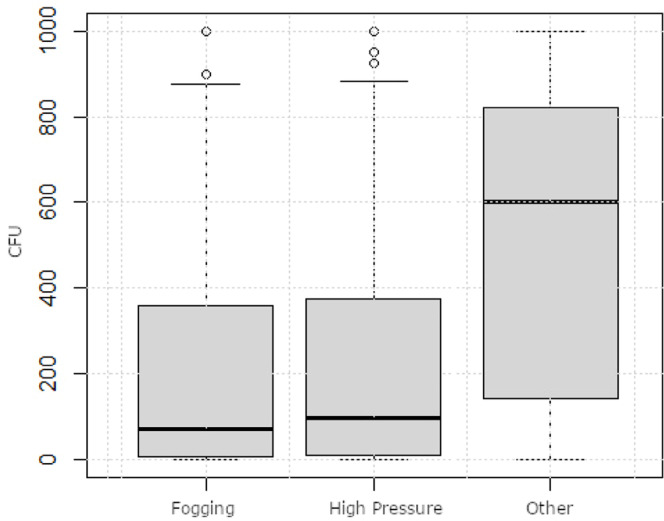
Distribution of bacterial counts presented as colony forming units (CFU) in dip slide samples from 1440 control points sampled during 72 production rounds at 18 farms categorized by disinfection applied by fogging (*n* = 48), high pressure washing (*n* = 17), and other (*n* = 7). Boxes show values between the 25th and 75th percentiles with the median indicated by a line. Values below the 10th and above the 90th percentile are shown as circles.

The application method for disinfectants was found to have a notable impact on the observed bacterial load after C&D. Methods that involved the use of a dry smoke disinfectant or slaked lime milk spread over the floor and walls, included in “others” in Figure 5, were associated with significantly higher (*p* < 0.001) bacterial counts compared to more effective methods such as fogging and spreading by high-pressure.

**Fig. 6:**
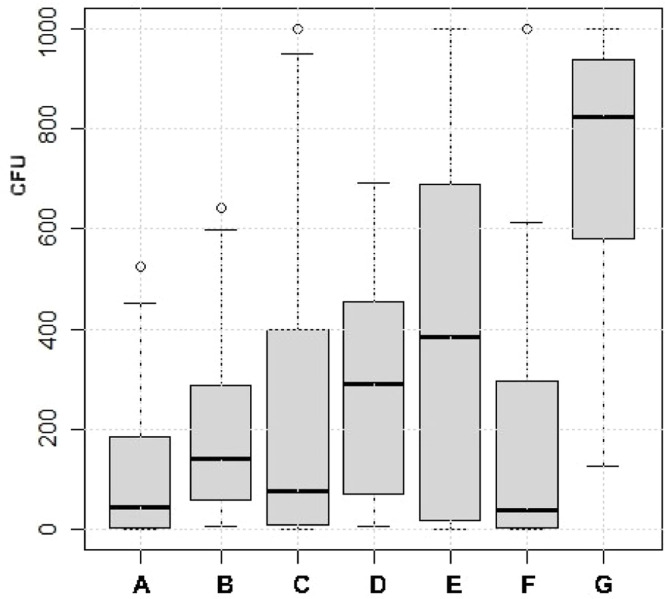
Distribution of bacterial counts presented as colony forming units (CFU) in dip slide samples from 1440 control points sampled during 72 production rounds at 18 farms after cleaning and disinfection with seven different disinfectants, hydrogen peroxide (A), peracetic acid (B), glutaraldehyde (C), quaternary ammonium substances (D), orthophenylphenol (E), formalin (F), and Ca(OH)_2_ (G). Boxes show values between the 25th and 75th percentiles with the median indicated by a line. Values below the 10th and above the 90th percentile are shown as circles.

In instances where the disinfectant used was a compound product, the group was categorized based on the main active ingredient. The most frequent (76 %) main active ingredient in the disinfection products was glutaraldehyde, followed by hydrogen peroxide (6 %), peratic acid (4 %), calcium hydroxide (4 %), formalin (4 %), and orthophenylphenol (4 %). In contrast, quaternary ammonium substances alone were used less frequently (1 %) (Fig. 6). No significant differences were observed in bacterial loads after C&D between the different disinfection products. However, it must be noted that certain products were only used in one production round, while the most used disinfectant was applied across 55 production rounds.

### Comparison of disinfectants

No significant differences could be observed regarding the effectiveness of different disinfectants, potentially because of uneven sample sizes for the various disinfectants. As a result, the study was extended to compare three commonly used disinfectants: hydrogen peroxide (A), peracetic acid (B), and glutaraldehyde combined with benzalkoniumchloride (C). Chlorine dioxide (H) was also included in the comparison.

**Fig. 7:**
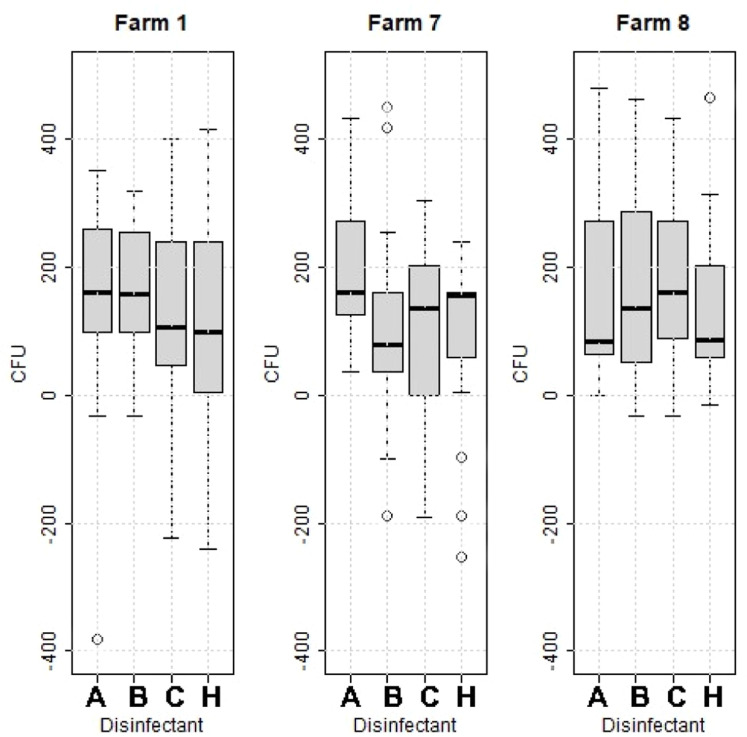
Reduction of bacterial load of 360 samples collected before and 360 samples collected after disinfection divided by four disinfectants: hydrogen peroxide (A), peracetic acid (B), glutaraldehyde combined with benzalkoniumchloride (C), and chlorine dioxide (H), during two sampling occasions at four compartments at three broiler producers. Boxes show values between the 25th and 75th percentiles with the median indicated by a line. Values below the 10th and above the 90th percentile are shown as circles.

All disinfectants effectively reduced the bacterial load. Although no substantial differences in efficacy were observed between the disinfectants, as all four tested substances showed similar mean reductions, ranging from 110 to 170 CFU per sampling point, there was a tendency for differences between farms. The bacterial reduction at Farm 1 differed from the others, with a reduction above 400 CFU observed on only one occasion, regardless of the disinfectant used. In contrast, at Farm 8, all four disinfectants achieved a reduction of more than 400 CFU per plate at least once for each disinfectant and consistently demonstrated less pronounced reductions in bacterial load across the different disinfectants (Fig. 7).

## Discussion

Cleaning and disinfection (C&D) of poultry houses is a complex process consisting of multiple steps, where each step has a potency to influence overall effectiveness. The absence of differences in bacterial load between different areas of the broiler houses in this study could be interpreted in various ways: either all surfaces were equally dirty from the outset, or they became equally contaminated after the cleaning process. Regardless, it must be emphasized that C&D should be performed thoroughly on all surfaces within the poultry house, including the feeders and drinking water lines. The significant differences in bacterial load after C&D between the farms in this study highlight that the execution of protocols and routines vary. Additionally, the range in bacterial loads after C&D on the same farm differed between sampling occasions, suggesting that achieving consistent results over time is challenging. A narrower range of bacterial loads on certain farms indicated better C&D routines and performance. This study also detected variations in bacterial counts after C&D across different farms. During the testing of the four different disinfectants on the farms included, one farm (Farm 8) showed a greater bacterial reduction, with a reduction of over 400 CFU at more control points compared to the other farms, and fewer instances of reductions below 0. Throughout the study period, *Campylobacter* could not be detected from any of the broilers delivered to slaughter from Farm 8, within the Swedish Campylobacter program (Hansson et al., 2007). This finding aligns with previous studies, which emphasize the importance of thorough cleaning by individual broiler producers for achieving an improved production outcome. These studies have shown that the overall cleanliness of broiler farms plays a significant role, with a significant connection between poor general tidiness and a higher prevalence of *Campylobacter* in chickens (Hansson et al., 2010; Newell et al., 2011). Additionally, Burbarelli et al. (2017) found that improvements in the cleaning program of broiler houses had a positive impact on the birds’ performance. Similar results have also been observed by Bragg and Plumstead (2003) who found that a comprehensive continuous disinfection program reduced mortality caused by infectious agents and decrease bacterial levels in broiler houses that underwent thorough continuous disinfection.

This study found that neither the time between C&D nor the duration of the empty period was correlated with the bacterial counts remaining after C&D. This is consistent with the findings of Luyckx et al. (2016) who observed that extending the vacancy period in pig nursery units to 10 days after disinfection had no impact on the environmental bacterial load.

A significant reduction in bacterial counts was observed when the surfaces were soaked with water before cleaning. This finding is in agreement with a study by Luyckx et al. (2015) which showed that overnight soaking with water led to a greater bacterial reduction compared to cleaning protocols without a preceding soaking step. For the soaking step, water should be finely misted so that it can penetrate the dirt. Additionally, that same study found no differences between protocols using cold or warm water during cleaning, which also aligns with the results of our study. Although hot water from a modern high-pressure washer can shorten cleaning time, it also accelerates the drying of surfaces, allowing disinfection, repairs, and other tasks in the broiler house to begin sooner.

The high-pressure cleaner has the additional advantage of dissolving greasy dirt quicker and effectively combats germs, even without the use of detergent agents. A study in the U.S. reported that a combination of pressurized steam followed by forced hot air was a more efficient cleaning procedure for transport crate floors compared to water washing, pressurized steam, or forced hot air alone (Reina et al., 2024). However, it is important to note that although the procedure reduced bacterial levels, pathogens such as *Salmonella* and *Campylobacter* were still detected in that study.

It is well-known that surfaces must be cleaned before disinfection to remove organic matter, and that the disinfectant have to be applied both at the required concentration and for the appropriate contact time (Langsrud et al., , 2003). In our study, no bacterial reduction calculation was made in relation to cleaning. The reason for this was due to differences in the design and layout of the broiler houses, which made comparisons between different producers unreliable. Further, there were variations in cleaning routines among the broiler producers, including factors such as the time spent cleaning, the concentration and active ingredients of the cleaning agent, the time between rough cleaning and final cleaning, soaking time, and other variables. For the comparison of the four disinfectants applied in identical broiler compartments, a cold fog resonator was used, which produced fine droplets that enhanced surface coverage. This allowed for more uniform distribution and better access to hard-to-reach areas compared to spraying and foaming, making it particularly effective in large spaces such as a broiler house. When the disinfectants were compared in identical broiler compartments, each cleaned according to the same protocol, no significant differences were found between them, as all disinfectants displayed a mean reduction of 2.1 to 2.2 log CFU. This reduction could be considered relatively reasonable; in a study on cleaning and disinfection in a pig nursery unit, the greatest reduction of total aerobic bacteria was 1.6 log CFU, measured four days after disinfection (Luyckx et al., 2016). In contrast, Battersby et al. (2017) achieved a reduction in total viable count ranging from 0.7 to 3.2 log10 CFU/m² using hydrogen peroxide disinfection by spraying, and a significantly higher reduction of 4.3 to 6.0 log10 CFU/m² when fogging with a Glutaraldehyde-Quaternary ammonium complex.

The most frequently used active components in disinfectants were a combination of glutaraldehyde and quaternary ammonium compounds. Formaldehyde and a combination of peracetic acid and hydrogen peroxide were also employed, but to a lesser extent. The results of this study revealed no evidence to suggest that the choice of disinfectants post-cleaning affected bacterial load, contrasting to a study by Carrique-Mas et al. (2009) on C&D performed in the UK, which focused on the elimination of *Salmonella* spp. in laying hens. They observed that 10 % formalin resulted in a greater reduction of *Salmonella* in laying hen flocks compared to a mixture of formaldehyde, glutaraldehyde, and quaternary ammonium compounds. However, the use of formaldehyde is not entirely ideal, as it should be applied by a specialist contractor for health and safety reasons (d’Ettorre et al., 2021). Disinfection carried out by a specialist contractor also offers additional benefits; a study in Belgium demonstrated better results when disinfection was performed by a contractor, rather than the farmer (Maertens et al., 2018). In another study examining the effectiveness of various disinfection methods to eliminate *Salmonella* contamination in turkey houses, a mixture of formaldehyde, glutaraldehyde, and quaternary ammonium compounds outperformed products containing hydrogen peroxide, and peracetic acid showed the best result (Mueller‐Doblies et al., 2010). Conversely, Marin et al. (2009) found that the use of glutaraldehyde, formaldehyde, and peroxygen at a concentration of 1.0 % in field conditions are inadequate for *Salmonella* elimination in the environment of layers irrespective of the serotype, the biofilm development capacity, and the disinfectant contact time. The sensitivity to C&D appears to vary across bacterial species. Certain bacteria, such as *Campylobacter* spp., appear to be sensitive to most C&D treatments. A study by Battersby et al. (2017) demonstrated that a combination of quaternary ammonium compounds and glutaraldehyde was effective in eliminating *Campylobacter* from most sites in broiler houses such as feeders, drinkers, walls, columns, barriers and bird weigh in broiler houses. However, bacteria belonging to the family *Enterobacteriaceae*, such as *Salmonella, Klebsiella* spp., and *E. coli*, were more challenging to eliminate. *Salmonella* spp. is relatively resistant to C&D procedures compared to most avian pathogens, and can survive for extended periods in residual organic matter in poultry houses (Andino and Hanning, 2015). Additionally, Robé et al. (2024) found that although C&D can reduce bacterial load, a complete elimination of ESBL- and pAmpC-producing *E. coli* does not seem achievable in broiler houses. Therefore, a multifactorial approach that combines various hygiene and management measures is required to reduce ESBL-/pAmpC-producing *E. coli* in broiler farms. In Belgium, Maertens et al. (2018) analyzed the efficacy of C&D by using hygienogram scores after treatment in nearly 20,000 poultry flocks in Flanders. The combination of peracetic acid and hydrogen peroxide, or formaldehyde, resulted in the best scores. In contrast, disinfection with formaldehyde alone or with disinfectants containing both peracetic acid and hydrogen peroxide yielded the lowest hygienogram scores.

## Conclusion

Effective cleaning and disinfection are fundamental to reducing pathogenic bacteria in broiler houses. To maximize their impact, these practices should be optimized by incorporating pre-soaking, detergent use, and thorough disinfectant application. Techniques such as fogging and high-pressure washing further enhance bacterial reduction. However, the success of these measures is not solely dependent on the methods used but also on factors such as operator technique and environmental conditions. Therefore, training and adherence to standardized protocols are critical to ensure consistent and effective biosecurity in broiler production. Ultimately, the success of cleaning and disinfection seems largely dependent on the performance of the operators carrying out the process.

## Declaration of competing interest

The authors declare that they have no known competing financial interests or personal relationships that could have appeared to influence the work reported in this paper.
